# Maternal undernutrition does not alter Sertoli cell numbers or the expression of key developmental markers in the mid-gestation ovine fetal testis

**DOI:** 10.1186/1477-5751-12-2

**Published:** 2013-01-08

**Authors:** Luis P Andrade, Stewart M Rhind, Michael T Rae, Carol E Kyle, Jamie Jowett, Richard G Lea

**Affiliations:** 1 Instituto Politécnico de Castelo Branco, Escola Superior Agraria, Quinta Sr.ª Mércules, Castelo Branco, 6001-909, Portugal; 2 The James Hutton Institute, Craigiebuckler, Aberdeen, AB15 8QH, UK; 3 Edinburgh Napier University, 10 Colinton Road, Edinburgh, EH10 5DT, UK; 4 School of Veterinary Medicine and Science, University of Nottingham, Sutton Bonington Campus, College Road, Sutton Bonington, Loughborough, LE12 6RD, UK

**Keywords:** Testis, Sertoli, Undernutrition, Fetal, Sheep, Gonad

## Abstract

**Background:**

The aim of this study was to determine the effects of maternal undernutrition on ovine fetal testis morphology and expression of relevant histological indicators. Maternal undernutrition, in sheep, has been reported, previously, to alter fetal ovary development, as indicated by delayed folliculogenesis and the altered expression of ovarian apoptosis-regulating gene products, at day 110 of gestation. It is not known whether or not maternal undernutrition alters the same gene products in the day 110 fetal testis.

**Design and methods:**

Mature Scottish Blackface ewes were fed either 100% (Control; C) or 50% (low; L) of estimated metabolisable energy requirements of a pregnant ewe, from mating to day 110 of gestation. All pregnant ewes were euthanized at day 110 and a sub-set of male fetuses was randomly selected (6 C and 9 L) for histology studies designed to address the effect of nutritional state on several indices of testis development. Sertoli cell numbers were measured using a stereological method and Ki67 (cell proliferation index), Bax (pro-apoptosis), Mcl-1 (anti-apoptosis), SCF and c-kit ligand (development and apoptosis) gene expression was measured in Bouins-fixed fetal testis using immunohistochemistry.

**Results:**

No significant differences were observed in numbers of Sertoli cells or testicular Ki67 positive cells. The latter were localised to the testicular cords and interstitium. Bax and Mcl-1 were localised specifically to the germ cells whereas c-kit was localised to both the cords and interstitium. SCF staining was very sparse. No treatment effects were observed for any of the markers examined.

**Conclusions:**

These data suggest that, unlike in the fetal ovary, maternal undernutrition for the first 110 days of gestation affects neither the morphology of the fetal testis nor the expression of gene products which regulate apoptosis. It is postulated that the effects of fetal undernutrition on testis function may be expressed through hypothalamic-pituitary changes.

## Background

In sheep, maternal undernutrition during gestation compromises both structure and function of the post-natal testis. Specifically, maternal undernutrition (70 days of gestation to term) has been reported to reduce the number of Sertoli cells in the testes of newborn lambs [1]. In addition, placentally-mediated fetal growth restriction, induced by overfeeding adolescent ewes throughout gestation, delayed the seasonal increase in testosterone and reduced peak testosterone concentrations in pubertal offspring [2]. Female offspring of ewes subjected to undernutrition for the first 95 days of gestation, also exhibit effects on the reproductive system in the form of reduced ovulation rates [3]. Although the mechanism of this is uncertain, it may reflect earlier changes in the fetal ovary which have also been reported in fetuses of undernourished ewes [4]. In contrast to the ovary, little is known about causal relationships between the effects of maternal undernutrition on the fetal testis and possible post-natal consequences. However, such effects are of potentially great economic significance since fully fertile and active rams are critical for sheep flock productivity.

Various researchers have attempted to identify key, nutritionally-sensitive developmental stages of the fetal gonad. In the fetal ovary, all stages of gestation appear to be sensitive to maternal undernutrition including the 0–30 day period of gestation [5] before gonadal differentiation occurs [6]. With respect to the fetal testis, provision of a maternal diet meeting 70% of maintenance requirements from 70 to 120 days (approx 145 day gestation) reduced the number of Sertoli cells in 2 day old lambs [7]. Similarly, 10 month old lambs born to ewes fed a 50% maintenance diet from 31 to 100 days of gestation exhibited fewer Sertoli cells, a smaller seminiferous tubule diameter and an elevated FSH response to a GnRH challenge, relative to controls [8]. Intriguingly, in the same study, the FSH response was unaffected at 2 and 5.5 months after birth. Moreover, if the period of undernutrition was restricted to the first 30 days of gestation, prior to the period of sexual differentiation, no differences were observed at any of the 3 postnatal time points (2, 5.5 and 10 months). In another study, *in situ* hybridisation studies revealed that maternal undernutrition applied for the first 50 days of gestation (50% maintenance diet), increased the mRNA expression of a key rate-limiting factor in steroid synthesis, the cholesterol transporter, steroidogenic acute regulatory protein (*STAR*) [9]. This was accompanied by increased fetal testosterone concentrations, indicating altered endocrine function. This effect was transient since it was not detected 15 days later, at day 65. Collectively, these observations suggest that the mechanisms that control fetal testis development are complex and time-dependent and that effects exerted during gestation may only be apparent in later life. It is uncertain whether or not such early changes in fetal testicular endocrine function are linked to observed changes in testis structure and function at puberty.

In order to gain understanding of these apparently anomalous observations, there is a need to characterise associated, nutritionally-mediated changes in developmental genes and associated effects. Normal testis development after differentiation, which is complete by 80 to 100 days of gestation, depends upon the appropriate regulation of cell turnover, proliferation and death [10-12]. These processes are regulated by genes expressed in the fetal testis and include those of the bcl-2 family such as the pro-apoptotic factor, Bax, and its antagonists Mcl-1 and Bcl2 [12-14]. Bax has previously been localised to human fetal Sertoli cells [14] and has a crucial role in testis development [15]. Mcl-1 is present in human fetal Leydig and Sertoli cells and in fetal ovine oocytes at all developmental stages [16]. In addition, fetal ovarian Mcl-1 protein is responsive to both maternal undernutrition and to exposure of the pregnant ewe to environmental concentrations of endocrine disrupting chemicals [4,16].

The stem cell factor (SCF)/c-kit ligand receptor system has been implicated, also, in the regulation of apoptosis in testicular germ cells [17,18]. Indeed, rat, *in vivo* studies indicate that SCF plays a role as a pro-survival factor for mature Leydig cells and, in a study where Leydig cells were experimentally depleted with ethylene dimethane sulphate (EDS), it was shown that SCF also plays a role as a growth factor for precursor Leydig cells [19]. SCF has been identified, previously, in fetal ovine interstititial cells [10] and this ligand receptor system has been implicated in the regulation of germ cell migration and spermatogenesis [20,21]. Consequently, the measurement of SCF and c-kit genes is relevant to investigations into the mechanisms by which previously reported effects of undernutrition may be exerted.

In addition to the above genes which primarily regulate apoptosis, indices of proliferation are also important measures of tissue development. In this regard, the Ki67 protein is recognised as an excellent marker of cell proliferation [22], being present during all active phases of the cell cycle, and absent in resting cells.

We have shown, previously, that maternal undernutrition delays ovarian folliculogenesis in the day 110 fetal ovary and increases the ovarian expression of the apoptosis-regulating gene products, Mcl-1 and Bax [4,23]. Since underfeeding dams also reduces ovulation rates in adult offspring [3], it is possible that there is a causal relationship between these two sets of observations. In view of previous reports of compromised testicular structure in adult sheep born to nutritionally-restricted dams, we hypothesised that an early driver of these changes may be a reduction in fetal testis Sertoli cell numbers and/or changes in developmental gene expression similar to those observed in the ovaries of fetuses from undernourished ewes. Consequently we examined day 110 fetal testes from undernourished ewes for Sertoli cell numbers, cell proliferation and the expression of developmental genes shown, previously, to be altered in fetal ovaries at the same stage of gestation.

## Results

### Maternal body condition scores

By 110 days gestation, the mean (± SEM) body condition score of the C ewes (n = 11) had changed little (2.46 ± 0.02 at mating; 2.41 ± 0.04 at 110 days) but that of the L ewes (n = 19) had declined (2.46 ± 0.02 at mating; 1.93 ± 0.05 at 110 days).

### Fetal and testis weights

A male fetus was collected, from each of the 6 C and 9 L ewes carrying one or more male fetuses (see materials and methods). Mean (± SEM) male fetal weights (g) were greater in C than L animals (C: 2200 ± 77 v L: 1862 ± 58; P < 0.01) but mean (± SEM) fetal testis weights (mg) were similar in the two treatments (C: 497 ± 42 v L: 449 ± 24, NS).

### Sertoli cell numbers and cell proliferation in the fetal testis

**Figure 1:**
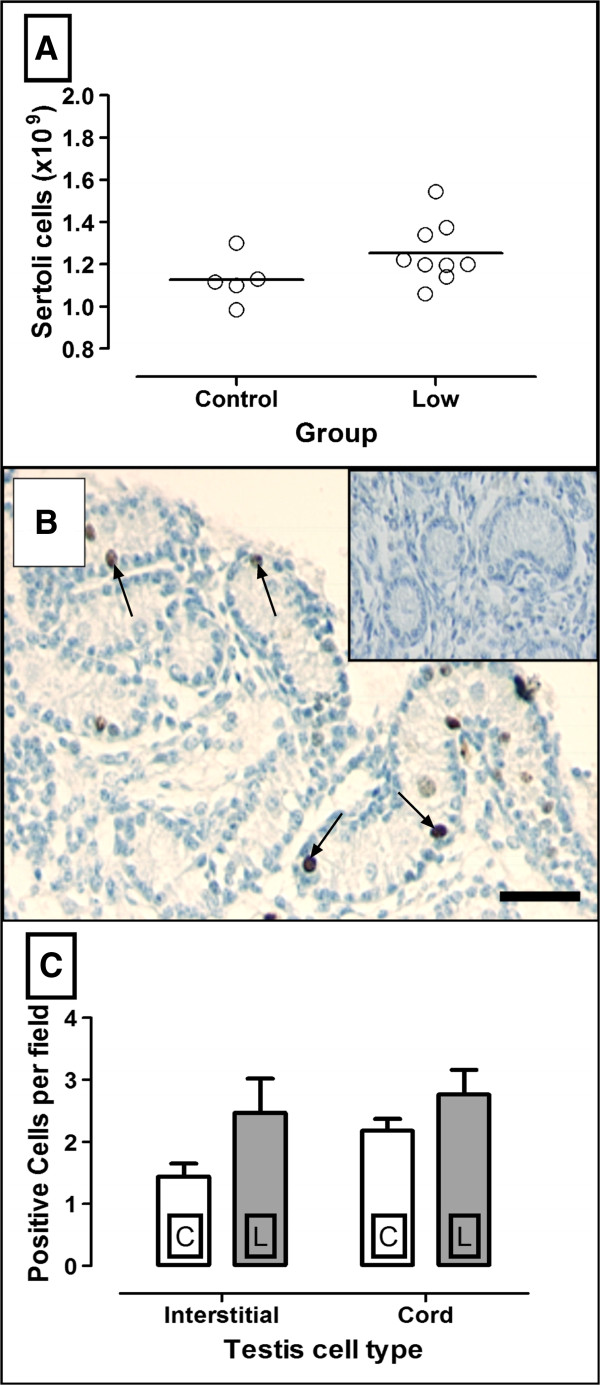
**Sertoli cell numbers and the expression of Ki67 in testes of day 110 sheep fetuses from ewes fed 100% maintenance requirements (control C: n = 6) or 50% maintenance requirements (Low; L; n = 9) diets.** (**A**) Fetal testis Sertoli cell numbers, (**B**) localisation of the proliferation marker Ki67; arrows depict positive Ki67 nuclear staining (**C**) Numbers of Ki67 positive cells counted in the seminiferous cords and in the interstitial area. No significant differences were observed between C and L testes. Values are expressed as means ± SEM. In B, scale bar = 50 μM. Inset = IgG negative control.

Numbers of Sertoli cells in fetal testes from underfed mothers (n = 9) were not significantly different to those of the control group (n = 6) (Figure 1A: P = 0.08). Ki67 was predominantly localised to the testicular cords with staining most obvious in some Sertoli and germ cells. Some positive-staining cells were observed in the interstitium (Figure 1B). C (n = 6) and L (n = 9) animals exhibited no significant differences in numbers of Ki67-positive cells in either the cords or interstitial areas of the fetal testis (Figure 1C).

### Fetal testicular Mcl-1 and Bax

**Figure 2:**
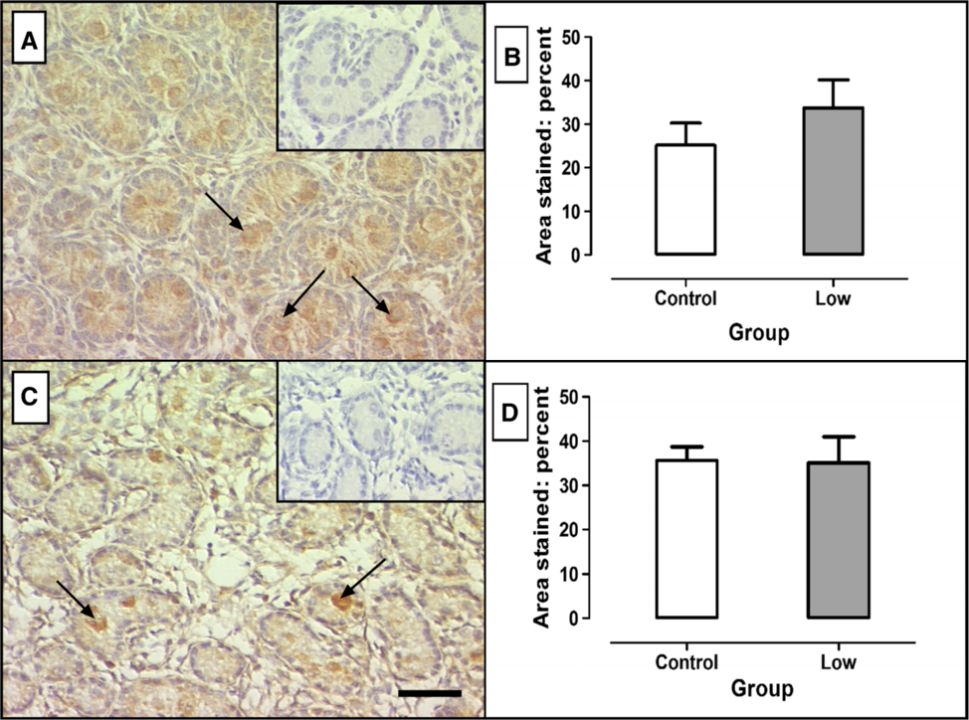
**Localisation and percentages of nucleated areas stained for Mcl-1 (A,B) and Bax (C,D) in day 110 fetal testes.** Mcl-1 (anti-apoptosis) (**A**) and its antagonist Bax (**C**) are predominant in the germ cells (arrows). No significant differences were observed between testes from C ewes (100% maintenance diet: n = 6) and L ewes ( 50% maintenance diet: n = 9) ewes. Scale bar = 50 μM. Inset = IgG negative control. Values are expressed as means ± SEM.

In the day 110 fetal testis, Mcl-1 was predominantly localised to the germ cells in the seminiferous cords with light staining in the Sertoli cells and interstitial areas (Figure 2A). Bax was also predominantly localised to the germ cells in the testicular cords with very light interstitial staining in some areas (Figure 2C). C (n = 6) and L (n = 9) animals exhibited no significant differences in the percentage of testicular area stained for Mcl-1 (Figure 2B) or Bax (Figure 2D).

### Fetal testicular SCF and c-kit

**Figure 3:**
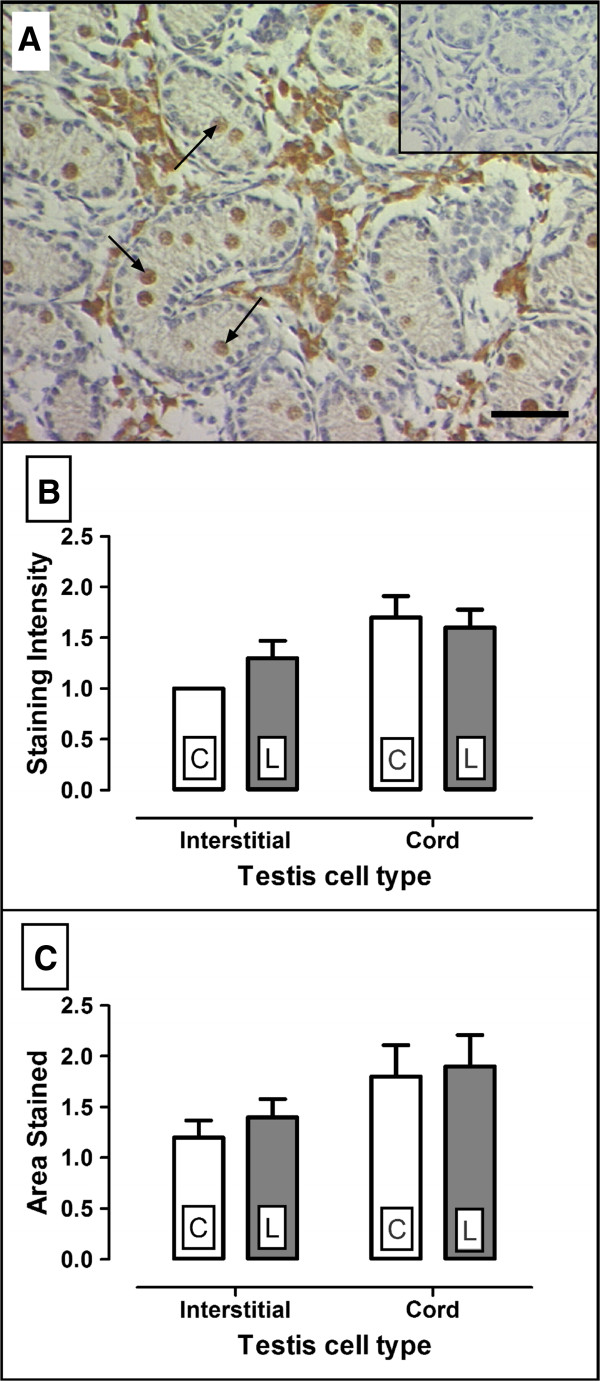
**Localisation and semi quantification of c-kit ligand staining in fetal testes collected from ewes on 100% maintenance (control: C, n = 6) and 50% maintenance (L) diets (n = 9).** c-kit (**A**) was localised to the cord germ cells (arrows) and interstitial area. Staining intensity (**B**) and area stained (**C**) were visually assessed by a single observer using an arbitrary four point scale. The cords and interstitial areas were assessed independently. No significant differences were observed between C and L testes. Scale bar (**A**) = 50 μM. Inset (**A**) = IgG negative control. Values (**B,C**) were expressed as means ± SEM. Where no error bar is given, all visually assessed values were identical.

C-kit was localised to the cord germ cells and interstitial areas of the testis (Figure 3A). Staining intensity in the seminiferous cords and interstitium (Figure 3B) and area stained in these same regions (Figure 3C) were similar in C (n = 6) and L (n = 9) testes. In contrast, SCF immunostaining was sparse with only light staining in the interstitium (not shown) which was too pale to be quantified.

## Discussion

In an earlier study, male fetuses from pregnant ewes fed 50% of liveweight maintenance requirements from day 0 of gestation exhibited reduced fetal weight at day 110 of gestation [9] but no reduction in testicular size; the results of the current study confirm these previous observations. In contrast, day 110 female fetuses, from the earlier study, showed no treatment effect on either fetal weight or mean ovary weight [23]. The impact of maternal undernutrition on male fetal weight probably reflects the inability of the ewe to respond to the increasing nutrient demand of the faster-growing fetus, at this stage of gestation. The mean loss of 0.5 condition score units in the dams of the current study subjected to restricted nutrition, from 0 to 110 days, is consistent with that known to occur in poorly nourished animals, in practice, and is indicative of the nutritional demands on the dams.

The observation that the proliferation marker (Ki67) is localised to Sertoli and germ cells in the day 110 fetal testis supports our previous published study using another marker of proliferation (proliferating cell nuclear antigen: PCNA) which localised to the same testicular cell types [10]. Ki67 is a better marker of cell proliferation than PCNA since it is expressed at all stages of the cell cycle and is rapidly degraded when proliferation ceases, unlike PCNA which has a half-life of 20 hours and is also involved in DNA repair [24,25].

The localisation to germ cells of both the pro-apoptotic factor, Bax, and its antagonist Mcl-1, indicates that these factors play a role in the control of germ cell apoptosis. Indeed, apoptosis is reported to play a crucial role in both the control of germ cell numbers and elimination of defective germ cells during development [12]. Since the homodimerisation or heterodimerisation of Mcl-1 with Bax determines the overall effect on apoptosis, the ratio of these factors in germ cells is likely to determine germ cell survival. The c-kit/SCF system is also critical for germ cell survival; mouse knock outs for either of these factors result in testes devoid of germ cells [26,27] and other mouse, *in vitro* studies have shown that the interaction between germ cell c-kit and SCF is required for proliferation and migration of germ cells (reviewed in [28], [20]). In addition, murine in vitro studies indicate that the effects of SCF-c-kit interaction on germ cell survival operate through the Bcl-2 family [18].

In the current study, although c-kit, Bax and Mcl-1 were detected in testicular germ cells, these genes were not affected by maternal undernutrition. In contrast to the fetal testis, fetal ovarian development was shown to be perturbed in previous studies using the same experimental sheep model (50% maintenance feeding regime). The change was in the form of a delay in ovarian folliculogenesis at day 110 and this was associated with reduced germ cell proliferation at day 65 and an increased expression of Bax in granulosa cells at day 110 [4,23]. Since there was no effect of maternal undernutrition on these genes in the fetal testis, it is concluded that they are nutritionally-sensitive only in the developing fetal ovary.

We have reported, previously, that the testes of fetuses from underfed ewes exhibit a transient increase in the expression of the cholesterol transporter, *STAR,* at day 50 and that maternal undernutrition reduces GnRH sensitivity at day 115 [9,29]. This suggests that the timing of the nutritional insult may be crucial and/or that other components of the hypothalamic pituitary gonadal axis may be affected. It is possible, also, that other testicular genes not included in this study may be perturbed. However, given that the same nutritional treatment perturbed the expression of Ki67, Bax and Mcl-1 in the day 110 fetal ovary [4] and that the markers are related to proliferation, apoptosis and development, we suggest that there are few other likely candidate genes at this stage of gestation. However, this does not rule out the possibility of effects on these genes, or alternative genes, in the developing testis later in gestation or during the post-natal period, or the expression of a transient effect such as that seen at an earlier stage of gestation [9].

In keeping with the lack of an effect on developmental genes, the current study indicates that maternal undernutrition for the first 110 days of gestation has no effect on Sertoli cell number. The 0 to 110 day window of exposure to maternal undernutrition encompasses sexual differentiation (day 30), the onset of pituitary function and gonadotrophin secretion (day 80) and a substantial part of the period of Sertoli cell proliferation which occurs from 70 days through to parturition [10,11]. Our data indicate, for the first time, that developmental stages falling in this gestational period remained largely unaffected in terms of fetal testis Sertoli cell numbers and testicular developmental gene expression. This is consistent with our previous observations of the effects of maternal undernutrition, from mating to day 95 of gestation, on testis size in male offspring at 6 weeks or 10 months of age [3]. In contrast, others have reported that underfeeding of the ewe from 70 days to parturition reduced Sertoli cell numbers and the absolute volume of cords in the newborn lamb [1] while maternal feed restriction from 31 to 100 days of gestation reduced Sertoli cell numbers and seminiferous cord size in 10 month old lambs [8]. These apparently conflicting results may reflect the developmental stage investigated. In the current study, in which the effects of maternal undernutrition were examined only on the pre-natal day 110 testis, the possibility remains of post-natal expression of pre-natal undernutrition effects on testicular structure or function.

Support for the concepts of action at the hypothalamic level and expression of effects at later developmental stages is provided by findings based on the adolescent sheep model (2x maintenance diet throughout gestation; placental growth restriction and reduced lamb birth weight) [30]. As in the underfeeding model, testes from day 103 fetuses showed no changes in Sertoli cell numbers or the number of seminiferous cords [30] whereas pubertal lambs of 28 to 35 weeks of age from the same experimental model, had reduced testosterone concentrations and testicular volume and a delayed seasonal increase in testosterone [2]. Similarly, 20 month old male offspring from ewes fed a 50% maintenance diet from mating to day 95 of gestation exhibited increased FSH levels [3] and, in a separate study, maternal undernutrition from 31 to 100 days increased the FSH response to a GnRH challenge in 10 month old lambs [8]. Collectively, these findings suggest that fetal undernutrition may impact on the expression of genes which regulate the onset of postnatal hypothalamic-pituitary activity at puberty. The finding that suppression of the ovine pituitary testis axis during fetal life with a GnRH agonist reduces plasma testosterone concentrations in 28 week old lambs [31] supports this suggestion.

## Conclusions

Our data indicate that despite the exposure of the developing fetal testis to a nutritionally restricted environment, from conception to 110 days, fetal testis Sertoli cell numbers and testicular developmental gene expression were largely unaltered. We suggest that the fetal testis is less sensitive than the ovary to nutritional perturbation at this stage of pregnancy and conclude that there is a need to study, further, the impact of undernutrition on hypothalamic and/or pituitary function.

## Methods

### Animal management and nutritional treatments

All experimental procedures have been described elsewhere [23]. In brief, experiments involving live animals were conducted under the authority of the UK Animals (Scientific Procedures) Act, 1986, after Home Office and local ethics committee approval (The James Hutton Institute, Aberdeen). Mature Scottish Blackface ewes with a mean live weight (± SEM) of 59.3 ± 0.74 kg were mated at a synchronised oestrus (day 0 of gestation) after treatment, for 14 days, with intravaginal progestagen pessaries (Chronolone, 30 mg; Intervet, Cambridge, UK). At the time of mating, ewes were allocated randomly, within body condition score class (range 2.25 – 3.00, on a scale of 0 to 5 [32]), to one of two nutritional treatment groups. This widely used measure comprises a subjective assessment of fat cover on the back of the animal and is highly correlated with body fat reserves. Since the live weight of animals is greatly influenced by gut fill in studies involving nutritional manipulations such as this, body condition scores were considered to be a more useful measure of medium term changes in nutritional state.

The animals from which the experimental sub-sets were selected, randomly, were housed under natural day length conditions with access to water, *ad libitum,* and fed in individual pens as follows: normal, control intake (C) – liveweight maintenance ration (100% M) from mating to 110 days of gestation (n = 11); low intake (L) – 50% M from mating to 110 days of gestation (n = 19). From day 80 of gestation, amounts of feed were increased according to stage of pregnancy and treatment group [33]. The diet consisted of pelleted feed (Green Keil, North Eastern Farmers Ltd, Aberdeen, UK) and hay, that provided, initially, 8.0 MJ ME per day (C) or 4.0 MJ ME per day (L). Ewes were scanned at day 80 of gestation, using ultrasonography, to determine the numbers of fetuses and rations were then increased, as necessary, to maintain the same nutritional states i.e. through these adjustments, the nutritional state of the fetuses was similar, irrespective of whether they were singles or twins.

The effects of position in the uterus, and sex of co-twin, if present, were considered likely to be small and only demonstrable with very large numbers of animals. In the C group, there were 16 fetuses (8 singles and 4 twins) and in the L group there were 30 fetuses (8 singles and 11 twins). One male fetus was selected, randomly, from each of 6C and 9 L ewes where a male fetus was present. Samples were derived from single and twin litters (C: 4 single, 2 twin, L: 2 single, 7 twin). Of the 2 C twin pregnancies, one co-twin was male and of the 7 L twin pregnancies, 2 were male. Other fetuses were used for different studies [23].

### Tissue collection and processing

At day 110 of gestation, ewes bearing twin or single fetuses were euthanased with a lethal dose of barbituate anaesthetic (Euthatal: 500 mg/ml, 30 ml, i.v., Rhone Merieux, Harlow, UK). Male fetuses were recovered and weighed. Fetal testes were removed, weighed and immersion-fixed in Bouins solution (Sigma-Aldrich Company Ltd., Poole, UK) for 5.5 h. They were then rinsed and stored in 70% ethanol before being dehydrated, cleared and embedded in paraffin wax by standard methods, sectioned to 5 μm, and mounted on poly-L-lysine coated glass slides (Sigma-Aldrich Company Ltd.) prior to immunohistochemical analysis.

### Sertoli cell stereology

Sertoli cell numbers were determined using a stereological method based on that reported by Paul and colleagues in 2005 [34]. In brief, 5 μm sections were stained with haematoxylin Z (Cellpath plc, Hemel Hampstead, UK) and, from each section, 40 images were taken (10 from each pole) using a magnification of x 630. The software Image Pro Plus (Media Cyberbnetics, Wokingham, Berkshire) was used to generate a grid consisting of 432 evenly distributed points. This was superimposed over each digital image and the number of intersections on the grid overlying a Sertoli cell nucleus was counted using a manual tag system. The ‘total point count’ for Sertoli cells obtained was then expressed as a percentage of the maximum count possible (40 images x 432 possible intersections = 17,280) to give the ‘total point count percent’. This value was used to calculate the weight of testis made up of Sertoli cells and subsequently converted to volume occupied by Sertoli cells (absolute volume: AV) assuming 1 g of testis = 1 cm^3^[34]. Image pro plus (Media Cyberbnetics) was then used to measure the diameter of 2 Sertoli cell nuclei per image. Since Sertoli cell nuclei are not spherical, the program took the average length of several diameters. The mean nuclear volume (MNV) of each Sertoli cell was calculated from the diameter as 4/3Пr^3^. The total number of Sertoli cells per testis was then calculated AV(μm^3^)/MNV(μm^3^)] and adjusted for testis weight so that values were expressed as number of Sertoli cells per gram of testis.

### Histology and immunocytochemistry

All tissue sections were de-waxed in Histoclear (National Diagnostics, Hessel, Hull, UK), re-hydrated through a graded ethanol series (100%, 95%, 70%) and washed in Tris-buffered saline (TBS; 0.1 M Tris–HCl; pH 7.6; 0.85% NaCl) for 2 x 5 min. Antigen retrieval procedures were necessary for exposure of all epitopes and this was achieved by microwaving sections in 0.01 M citrate buffer (pH 6.0) on full power (100%, 700 Watt) for 3 × 5 min. Sections for both studies were placed in a DAKO Autostainer (DakoCytomation, Ely, UK) and incubated with the appropriate primary antibody for 30 minutes. Primary antibodies were as follows: (a) monoclonal mouse anti human Ki67 at a 1:100 dilution (Clone MIB-1: DakoCytomation, Ely, UK), (b) polyclonal rabbit anti-Bax at a 1:50 dilution (Santa Cruz Biotechnology, Inc., Heidelberg, Germany), (c) polyclonal rabbit anti-Mcl-1 at a 1:50 dilution (Serotec, Oxford, UK) (d) rabbit anti-human c-kit (polyclonal C-19 raised against carboxy terminal domain of human c-kit: Santa Cruz Biotechnology Inc, Santa Cruz, CA), at a 1:40 dilution (stock 200 μg ml^-1^) and (e) rabbit anti-ovine SCF (kindly supplied by Dr. Ken McNatty, Wallaceville Animal Research Station, Upper Hutt, New Zealand), at a 1:450 dilution. Negative controls were performed by replacing the primary antibodies with non-specific mouse or rabbit IgG. Antibody binding was visualised using the ChemMate peroxidase/DAB detection system (DAKO, Ely, Cambridgeshire, UK) and all sections were counterstained using haematoxylin Z (Cellpath plc, Hemel Hampstead, UK).

### Quantification of immunostaining

The immunohistochemistry results for Ki67, Bax and Mcl-1 were quantified by computer-aided image analysis. The image analysis system comprised an Olympus Corp. microscope (x 20 objective; New Hyde Park, NY) and Hamamatsu digital camera (Hamamatsu, Bridgewater, NJ) connected to a computer running Image-Pro Plus software (Media Cyberbnetics). Quantification by image analysis was conducted over six randomly selected fields of view, after which the mean and standard error had stabilised. The total area of positively stained cells (brown colour) was measured and expressed as a percentage of the total cellular area. Immuno-histochemical staining intensity and area for c-kit were measured visually and semi-quantitatively using an arbitrary four point scale. A score of 3 indicated intense staining, 2: moderate staining, 1: some positive staining and 0 indicated no staining. Cord and interstitial areas were assessed independently and all analyses were carried out by the same operator.

### Statistical analysis

All analyses were based on male fetuses collected from 6 C and 9 L ewes: one fetus per pregnancy (see ‘animal management and nutritional treatments’). Image analyses data were analysed using Genstat software. Data were tested for normal distribution and groups compared by one-way or two-way ANOVA and standard post-hoc *t* tests where appropriate. Data not normally distributed were analysed using the Kruskall-Wallis one-way ANOVA and between-group comparisons by the Mann–Whitney U test.

## Competing interests

The authors declare that they have no competing interests.

## Authors’ contributions

LPA carried out the histological studies and contributed towards the analysis and JJ carried out the Sertoli cell counting. SMR, MTR and CEK developed the sheep model and assisted in providing the tissues. RGL and SMR designed, developed and coordinated the study. All authors read and approved the final manuscript.
